# Acceleration of Intended Pozzolanic Reaction under Initial Thermal Treatment for Developing Cementless Fly Ash Based Mortar

**DOI:** 10.3390/ma10030225

**Published:** 2017-02-24

**Authors:** Yang-Hee Kwon, Sung-Hoon Kang, Sung-Gul Hong, Juhyuk Moon

**Affiliations:** 1 Department of Architecture & Architectural Engineering, Seoul National University, 1 Gwanak-ro, Gwanak-gu, Seoul 08826, Korea; yanga1126@naver.com (Y.-H.K.); sglhong@snu.ac.kr (S.-G.H.); 2 Department of Civil and Environmental Engineering, National University of Singapore, 1 Engineering Drive 2, Singapore 117576, Singapore

**Keywords:** cementless mortar, pozzolanic reaction, thermal treatment, fly ash, silica fume

## Abstract

Without using strong alkaline solution or ordinary Portland cement, a new structural binder consisting of fly ash and hydrated lime was hardened through an intensified pozzolanic reaction. The main experimental variables are the addition of silica fume and initial thermal treatment (60 °C for 3 days). A series of experiments consisting of mechanical testing (compressive and flexural strength, modulus of elasticity), X-ray diffraction, and measurements of the heat of hydration, pore structure, and shrinkage were conducted. These tests show that this new fly ash-based mortar has a compressive strength of 15 MPa at 91 days without any silica fume addition or initial thermal treatment. The strength increased to over 50 MPa based on the acceleration of the intensified pozzolanic reaction from the silica fume addition and initial thermal treatment. This is explained by a significant synergistic effect induced by the silica fume. It intensifies the pozzolanic reaction under thermal treatment and provides a space filling effect. This improved material performance can open a new pathway to utilize the industrial by-product of fly ash in cementless construction materials.

## 1. Introduction

Ordinary Portland cement (OPC) is one of the most widely used manufacturing materials in the world. Due to the increasing demand for the construction of infrastructures, houses, and buildings, the production amount of OPC has been significantly increasing. However, its manufacturing process consumes large amounts of energy and emits carbon dioxide gas, one of the main gases causing the greenhouse effect [1,2]. For example, the cement industry is currently responsible for around 7% of global greenhouse gas emissions [3]. This urgent environmental issue has been gaining a lot of attention due to the increasing interest in sustainable development. Therefore, it is necessary to find OPC alternatives that have comparable material properties such as strength and durability, as well as allowing for sustainable development by emitting less carbon dioxide gas and consuming less energy during the manufacturing process [4,5].

As an alternative, there has been research focusing on replacing a part of OPC with fly ash or ground granulated blast-furnace slag [6,7]. Fly ash is being used as a supplementary cementitious material due to its pozzolanic or cementitious characteristics. The partial replacement with fly ash is known to improve workability and long-term strength and to reduce the heat of hydration, thereby resulting in less thermal cracking [8]. Some studies have presented the mechanical characteristics of high-volume fly ash concrete. Poon et al. reported on the mechanical and material properties of concrete with replacements of 25% and 45% fly ash [9]. They used a superplasticizer to obtain good workability with low water/binder (w/b) ratios of 0.19 and 0.24. As a result, they showed higher 28 days strength (106 MPa) than OPC mortar (97 MPa) in the case of a 25% replacement and w/b of 0.24. Huang et al. tried to increase the replacement ratio by using up to 80% fly ash type F and reported that it showed a similar or higher compressive strength after 28 days (with 40% replacement) and 91 days (with 60%–80% replacement) than that without fly ash replacement [10]. Atiş studied the replacement ratios of 50% and 70%. By mixing at various w/b ratios (0.29–0.4) and using a superplasticizer, it was concluded that a 50% replacement is achievable in regular concrete, but over 70% is not recommended, especially for the production of high-strength concrete [11]. Similarly, some studies reported the possibility of a successful replacement of high-volume fly ash, though a high volume of cement (over 50%) is still required [12,13,14].

Although fly ash is currently used as a supplementary cementitious material, there are several technical problems for utilizing it in construction materials without cement. For example, geopolymer concrete is being actively researched worldwide due to its environmental benefits, including almost zero embedded carbon dioxide emissions. Geopolymer concrete can typically be manufactured from industrial by-products such as fly ash or ground granulated blast-furnace slag or natural aluminosilicate minerals such as kaolinite [15]. It has been shown that cementless binder can be synthesized by alkali activation of industrial by-products or natural pozzolan [16,17,18]. It has also been reported that the geopolymer concrete has superior mechanical properties and durability, as well as fire resistance, even when compared with OPC-based concrete [19,20,21]. However, its activation process should be accompanied with high-temperature curing to attain suitable mechanical properties. This is an essential process for the strength evolution of fly ash-based geopolymer concrete [22,23]. In particular, fly ash with a low reactive calcium content has a slow reaction rate; thus, high-temperature curing is indispensable [24]. In addition, the chemical types and concentrations of the alkali activator play a key role in the geopolymerization process, so the selection of an activator is one of the most important design steps [25,26]. Most typical chemical activators are sodium hydroxide (NaOH), sodium silicate, and a mixture that uses both NaOH and sodium silicate together. However, from a practical point of view, several technical problems that include rapid setting time, toxicity (due to the high pH of the alkaline activators), high cost, and high-temperature curing prevent geopolymer concrete from being actively used in the construction industry [26,27]. Therefore, it is crucial to develop new fly ash-based construction materials that do not rely on a highly alkaline solution and are preferably curable at ambient conditions.

Our previous research suggests a new ternary mix design for kaolinite clay-based structural mortar [28]. Without using cement or a high-pH alkaline activator, the intended pozzolanic reaction from incorporated admixtures of silica fume (SF) and hydrated lime could successfully activate the natural kaolinite mineral for structural materials applications. With the addition of SF, a physical filling effect as well as an additional pozzolanic reaction increased the compressive strength by up to 200%–300% compared to a binder without SF [28]. As an extension of our previous research, here we attempt to use fly ash as the base material and investigate various material properties. Along with the intended pozzolanic reaction, the effect of the thermal treatment on the reaction and its impact on the material properties are investigated by a series of mechanical tests on compressive strength, flexural strength, and modulus of elasticity, and microstructural experiments including heat of hydration, X-ray diffraction (XRD), porosimetry, and shrinkage behavior.

## 2. Materials and Methods

### 2.1. Materials

**Figure 1:**
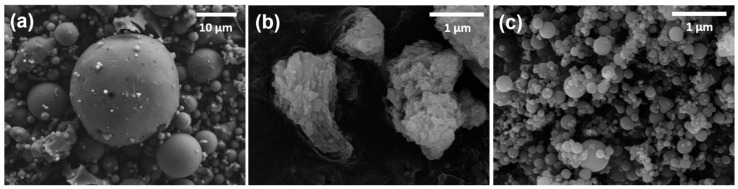
SEM images of raw materials: (**a**) fly ash; (**b**) hydrated lime and (**c**) silica fume.

**Figure 2:**
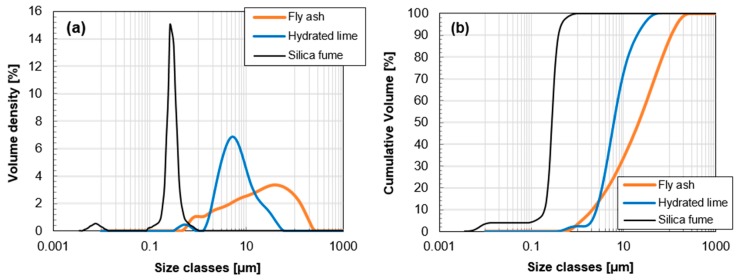
Particle size distributions of raw materials by laser diffraction particle size analysis: (**a**) volume density and (**b**) cumulative volume.

Table 1 shows the chemical compositions of the raw materials, which was determined by X-ray fluorescence (XRF) analysis using XRF-1700 (Shimadzu, Tokyo, Japan). Scanning electron microscope (SEM) images of the materials, obtained using JSM-7800F Prime (JEOL Ltd., Tokyo, Japan), are also presented in Figure 1. The raw fly ash used in this study was supplied from the Boryeong power plant, Boryeong, South Korea. As shown in Table 1, the main oxide components of the fly ash are SiO_2_ (61.7%), Al_2_O_3_ (18.7%), Fe_2_O_3_ (5.56%), and CaO (3.99%). Since the total value of SiO_2_ + Al_2_O_2_ + Fe_2_O_3_ is higher than 70% and the CaO component is less than 10%, the fly ash used is classified as Class F (low-calcium fly ash) according to ASTM C618 specifications [29]. Low-calcium fly ash (Class F) is predominantly composed of aluminosilicate glasses with varying amounts of crystalline quartz, mullite, and hematite. These crystalline phases are considered as inert phases in concrete. Used SF (SiO_2_ > 96.9%) has a density of 2.2 g/cm^3^ and a specific surface area of 200,000 cm^2^/g. The particle size distributions of raw materials that were measured by using Mastersizer 300 (Malvern Instruments, Malvern, UK) are presented in Figure 2. The median particle size (d_50_) is 39.8 μm for fly ash, 9.15 μm for hydrated lime, and 0.59 μm for SF. In order to maintain workability, a polycarboxylate ether-type superplasticizer was used together. The fine aggregate that satisfies ISO standards [30] was used in the production of all mortar mixtures.

### 2.2. Specimen Preparation

#### 2.2.1. Test Specimen

The mix proportion of the fly ash-based mortar is summarized in Table 2. Both the fly ash and hydrated lime are considered as binder materials. The main variables are the addition of SF to the mortar and the inclusion of steam curing to the curing program. The sample names in Table 2 are explained as follows: “amb” and “stm” indicate ambient curing and thermal treatment (including an initial 72 h of steam curing), respectively. The F+L samples are reference samples which do not include SF, while the F+L+SF samples contain 15% SF with respect to the weight of the binder. The water-to-binder ratio (w/b) for all samples was maintained at 25%. The ratio of fine aggregate-to-binder was determined to be 3, based on several preliminary tests.

All samples were mixed according to ASTM C305 [31] using a Hobart mixer. Firstly, dry materials such as fly ash, hydrated lime and fine aggregate (including SF) were blended for 30 s; the fine aggregate was excluded in the specimens used for hydration reaction tests such as heat of hydration and XRD, as it was assumed to be inert. Then, water and superplasticizer were added to the blended dry materials and mixed for 3 min at low speed (140 ± 5 rpm) and for another 1 min at high speed (285 ± 10 rpm). After mixing, the mortar was placed and compacted in the prepared molds. The specimens were wrapped with vinyl sheets to prevent moisture loss.

#### 2.2.2. Curing Program

The curing conditions were divided into two series: ambient curing (amb) and thermal treatment using steam curing (stm). The specimens with ambient curing such as F+L amb and F+L+SF amb were stored at a constant temperature (T = 20 ± 2 °C) and relative humidity (RH = 60% ± 5%) conditions until the test was performed. On the other hand, the thermal treatment (T = 60 ± 2 °C, RH = 95% ± 5%) was subjected only to the samples of F+L stm and F+L+SF stm for the first 72 h, in order to accelerate the hydration reaction. This condition is chosen based on a typical precast concrete production process. After the thermal treatment, all samples were cured in ambient conditions (T = 20 ± 2 °C, RH = 60% ± 5%) until subsequent testing.

### 2.3. Test Method

#### 2.3.1. Compressive Strength and Flexural Strength

The compressive strength and flexural strength were determined as the average of 3 measurements. According to ASTM C 109/C 109M [31], (50 × 50 × 50)-mm cubic specimens were loaded using a hydraulic universal testing machine at 1, 3, 7, 14, 28, 56, and 91 days. The flexural strength of a (40 × 40 × 160)-mm prismatic specimen was measured at 91 days according to the 3-point bending test method of ISO 679 [30]. 

#### 2.3.2. Elastic Modulus and Bulk Density

The elastic modulus and bulk density were also determined as the average of 3 measurements. To determine the modulus, a uniaxial compression with a constant displacement rate (0.1 mm/min) was subjected to the cylindrical specimens (Φ50 × 100 mm) at 91 days. The stress–strain curve was obtained from a load-cell and two strain gauges (PL–60 series, Tokyo Sokki Kenkyujo Co., Ltd., Tokyo, Japan) which were longitudinally and symmetrically attached on the surface of the specimens. Finally, the elastic modulus was calculated as the slope from the zero point to the 40%-of-maximum-stress point on the curve. Furthermore, to obtain the bulk density of the cylindrical specimens, the dimensions (diameter and height) and weights were measured before loading using a Vernier caliper (precision of 0.01 mm) and an electronic scale (precision of 0.01 g), respectively.

#### 2.3.3. Heat of Hydration

The heat of hydration was measured using an isothermal calorimeter (TAM AIR, TA Instruments, New Castle, DE, USA) for the first 3 days under the different temperatures of 20 °C and 60 °C. Before starting, 15 g of fresh paste was prepared and inserted into the 20 mL size glass bottle. For each measurement, the total heat of hydration of the fresh paste was calculated by integrating the heat flow curve. In order to allow for comparison among the different samples, the measured heat of hydration was divided by the weight of the binder in each paste.

#### 2.3.4. XRD Analysis

XRD analysis was performed to examine the mineralogical characteristics of all raw materials and hydrated samples. At 28 days of curing, the crushed and ground pastes were placed in a holder to perform the test. Each sample was scanned from 5° to 80° (2θ) with a step size of 0.0033° (Rigaku Miniflex, Tokyo, Japan). The crystalline phases were identified by comparing Bragg peak positions and intensities to those found in the Inorganic Crystal Structure Database (ICSD) database [32].

#### 2.3.5. Mercury Intrusion Porosimetry (MIP)

MIP was used to investigate the porosity and pore size distribution. On the 91st day, the crushed samples were dried at 105 °C. The test was conducted with the mercury parameter: 485 erg/cm^2^ for the surface tension and 130° for the contact angle. 

#### 2.3.6. Drying Shrinkage and Weight Loss

A free strain of (40 × 40 × 160)-mm prismatic specimen was recorded for 91 days, to measure drying shrinkage of each sample. When preparing the mortar specimen, a dumbbell-shaped strain gauge (PMFL-60 series, Tokyo Sokki Kenkyujo Co., Ltd., Tokyo, Japan) was embedded longitudinally in the center of the specimen, with the compaction of the mortar. The strain due to the drying shrinkage was recorded using a data-logger (TDS–530, Tokyo Sokki Kenkyujo Co., Ltd., Tokyo, Japan) every 5 min after the specimen was unsealed. This indicates that the strains of the specimens without steam curing (F+L amb and F+L+SF amb) were recorded from 1 day, while those of the specimens with steam curing (F+L stm and F+L+SF stm) were recorded from 3 days. During the same period of 91 days, the weight losses of the prismatic specimens were also measured using the electronic scale at one-day intervals to check the drying rate.

## 3. Results

### 3.1. Mechanical Properties

**Figure 3:**
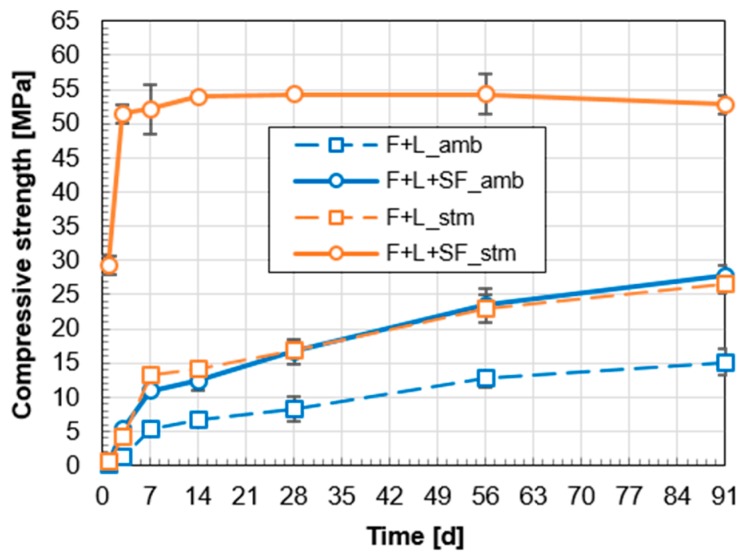
Compressive strength evolution of fly ash-based mortars.

Figure 3 demonstrates the successful development of a high-strength cementless fly ash-based mortar without using a highly alkaline activator. The compressive strength was remarkably increased with the addition of SF or thermal treatment of steam curing. Each treatment resulted in almost double strength, compared with the reference sample (F+L amb). Moreover, by applying thermal treatment and the addition of SF simultaneously, there was a significant improvement in the strength and its evolution rate. This is due to the synergistic effect of the pozzolanic reaction and its acceleration under increased temperature, which will be further discussed in Section 4.

**Figure 4:**
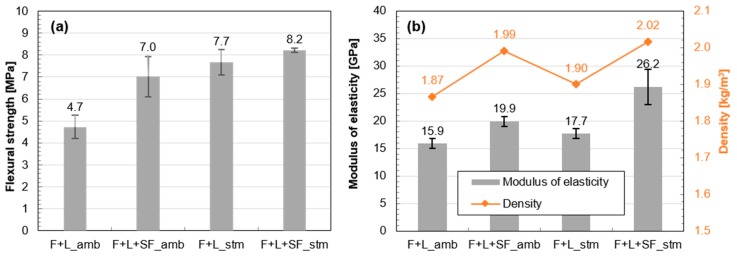
Mechanical properties of fly ash-based mortars at 91 days: (**a**) flexural strength and (**b**) modulus of elasticity and density.

The addition of SF or thermal treatment was also effective to increase the flexural strength, as shown in Figure 4a. Mechanical enhancement by applying both the addition of SF and thermal treatment simultaneously was not as remarkable as in the case of compressive strength. With regard to enhancing the modulus of elasticity, it was more effective to change the mix design by SF addition than by thermal treatment (Figure 4b). Similar to the compressive strength, applying both in conjunction increases the modulus. In addition, the density of the samples increased in a similar fashion to the increase in the modulus of elasticity. 

### 3.2. Hydration Reaction

#### 3.2.1. Heat of Hydration

**Figure 5:**
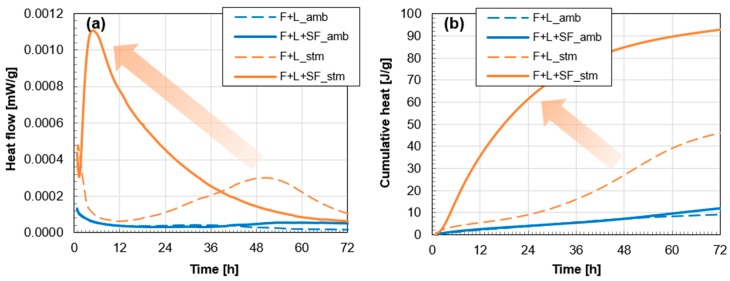
Hydration heat of fly ash-based on mortars: (**a**) heat flow curves and (**b**) cumulative heat curves.

The heat flow of the fly ash-based mortars differs significantly depending on the curing condition and SF addition. All samples show an immediate peak right after mixing, which then decreases (Figure 5a). This first peak is an indication of the dissolution of raw materials [33]. In the cases of either SF addition or thermal treatment, the heat flow was increased again. This additional heat flow indicates that the intensified pozzolanic reaction seemed to occur after 40 h in the F+L+SF amb sample. Furthermore, the thermal treatment accelerated and further intensified the pozzolanic reaction induced by the SF significantly. The F+L stm sample shows an initial induction period of up to 12 h, followed by an acceleration period of up to 50 h. On the other hand, F+L+SF stm significantly reduced the induction time to only 1.5 h, and then rapidly facilitated the acceleration period from 1.5–6 h. This result indicates the acceleration of the pozzolanic reaction due to the SF addition under thermal treatment. 

The cumulative heat of hydration presented in Figure 5b also supported the intensification of the pozzolanic reaction. Without the thermal treatment, the addition of SF barely accelerated the hydration reaction (compare blue lines in Figure 5b). It indicates that the majority (80%) of hydration heat was generated by the reaction of fly ash under ambient curing condition (see cumulative heat at 72 h). In other words, the reaction between the amorphous silica fume and hydrated lime was weak under ambient curing condition. However, the SF-induced reaction was significantly intensified during the thermal treatment period; it can be suggested that 50% of the heat was generated by the intensified SF-induced pozzolanic reaction under the treatment.

#### 3.2.2. XRD Analysis

**Figure 6:**
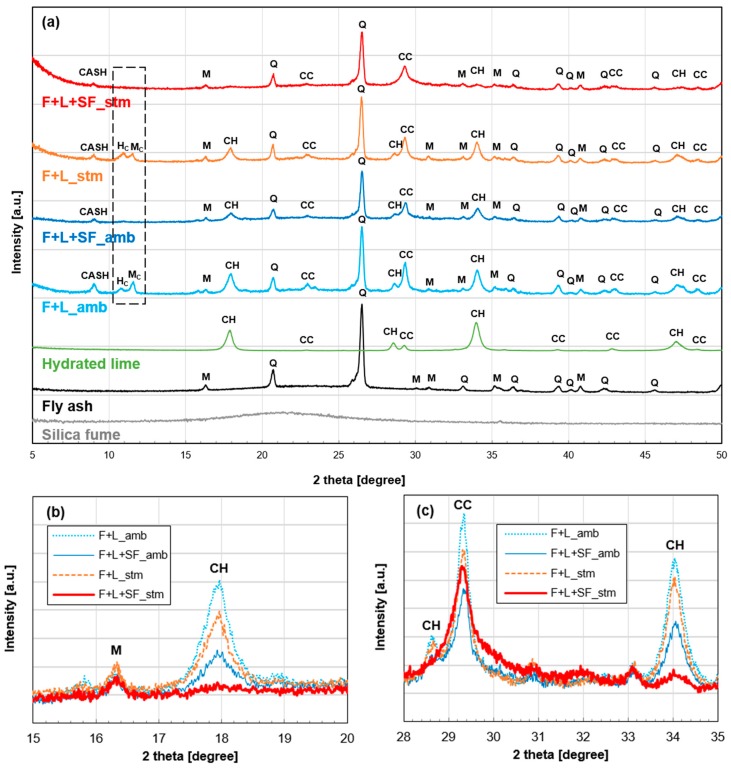
XRD patterns of (**a**) fly ash-based mortars and its raw materials; (**b**) calcium hydroxide and (**c**) calcium hydroxide and calcium carbonate: CASH = calcium aluminate silicate hydrate, CC = calcium carbonate (CaCO_3_), CH = calcium hydroxide (Ca(OH)_2_), H = hematite (Fe_2_O_3_), Hc = hemicarboaluminate (Ca_4_Al_2_(CO_3_)_0.5_(OH)_13_·5.5H_2_O), M = mullite (3Al_2_O_3_·2SiO_2_), Mc = monocarboaluminate (Ca_4_Al_2_(CO_3_)(OH)_12_·5H_2_O), Q = quartz (SiO_2_).

Figure 6a shows XRD patterns of all raw materials and hydrated samples at 28 days. Fly ash consists mostly of an amorphous phase (shown as a broad hump) and crystalline phases of mullite and quartz. The raw materials of hydrated lime contain calcium hydroxide (Ca(OH)_2_) and calcium carbonate (CaCO_3_), possibly due to ambient carbonation. Used SF did not show any crystalline phases. While all hydrated samples contain calcium aluminum silicate hydrate (CASH), only samples without SF (F+L amb and F+L stm) have carbonated Al_2_O_3_-Fe_2_O_3_-mono (AFm) phases of hemicarboaluminate (Ca_4_Al_2_(CO_3_)_0.5_(OH)_13_·5.5H_2_O) and monocarboaluminate (Ca_4_Al_2_(CO_3_)(OH)_12_·5H_2_O) (see black-dotted box in Figure 6a). This indicates that less carbonation occurred in samples with SF [34]. This can also be confirmed from the diminished intensity of the peak for calcium carbonate (at 29.4°) where there is SF addition (Figure 6c).

The peak intensity of Ca(OH)_2_ in F+L+SF amb was lower than that in F+L amb (Figure 6b,c). This is evidence of an additional pozzolanic reaction since the reaction consumes Ca(OH)_2_ [35]. In addition, by comparing the peaks of F+L amb and F+L stm, it is found that there is a decrease in the Ca(OH)_2_ peak without the SF addition. It can be explained that pozzolanic characteristic of fly ash consumes more Ca(OH)_2_ under thermal treatment. As expected, there is little remaining Ca(OH)_2_ in F+L+SF stm, where both the addition of SF and thermal treatment were applied together.

### 3.3. Pore Structure Analysis

**Figure 7:**
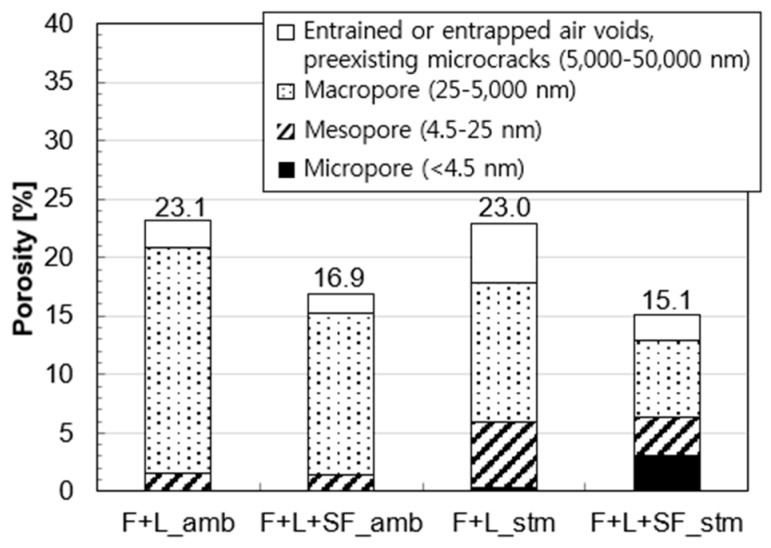
Porosity distribution of fly ash-based mortars at 91 days.

**Figure 8:**
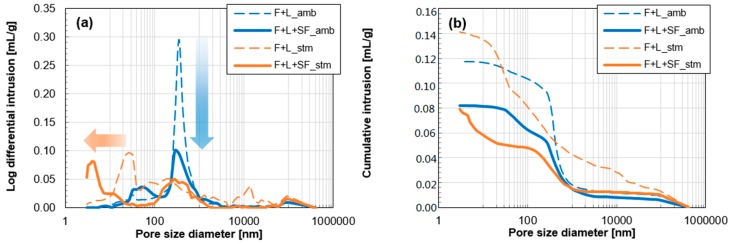
Pore size distribution of fly ash-based mortars at 91 days: (**a**) log differential intrusions and (**b**) cumulative intrusion.

Porosity analysis of the samples at 91 days is shown in Figure 7. For reducing the total porosity, the addition of SF was more effective than the thermal treatment. More specifically, Figure 8 shows the pore size distributions of the same samples. The main peak of the reference sample (F+L amb) was formed at 350 nm, and the intensity of the peak was decreased by 1/3 via SF addition as shown in Figure 8a. On the other hand, by applying the thermal treatment, the size of the main peak was decreased to 30 nm. This indicates that although the total porosity was not changed, the increased portion of mesopore volume remarkably contributed to the total porosity and resulted in a finer pore system. Furthermore, by using SF under thermal treatment, the main peak of F+L+SF stm was found at 5 nm. This also explains why there is a larger contribution of micropores in the total porosity, while other samples have smaller contributions.

### 3.4. Drying Shrinkage and Weight Loss

**Figure 9:**
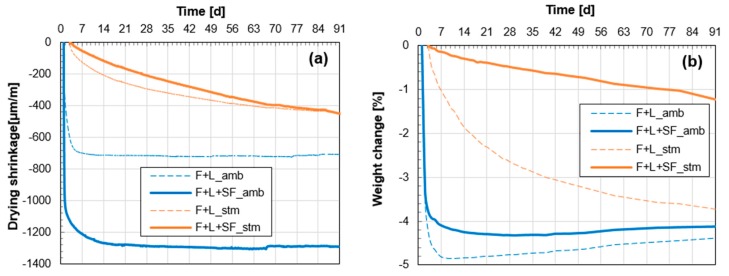
Variations of (**a**) drying shrinkage and (**b**) weight change of fly ash-based mortars.

Figure 9 shows the measured strains by drying shrinkage, and weight change for 91 days. At ambient conditions without the thermal treatment (blue lines), drying shrinkage occurred rapidly; the vast majority (>85%) of the total drying shrinkage occurred within the first 3 days. The addition of SF appears to accelerate the shrinkage. On the other hand, the drying shrinkage of the samples with the thermal treatment (orange lines) occurred slowly and continuously; the shrinkage did not converge even at 91 days. The strains of the thermally treated samples at this date were almost the same. However, the shrinkage rate at the early stage was slower when SF was added. 

The history of weight loss is presented in Figure 9b, which shows a similar trend with the shrinkage behavior (Figure 9a). While the weight loss of samples cured at ambient conditions (blue lines) converged at 7 days or 14 days, that of samples with the thermal treatment did not converge within 91 days. Unlike the drying shrinkage, the addition of SF did reduce the moisture loss, especially the specimens experiencing the thermal treatment. Meanwhile, it is interesting to note that the weight of ambient cured specimens actually increased after converging. This can be explained by the carbonation effect [36], as confirmed in the XRD analysis (Figure 6c). Carbonation occurs simultaneously with drying shrinkage [37]; thus, both weight loss due to evaporation and weight gain due to precipitation of calcium carbonate occurred concurrently.

## 4. Discussion

### 4.1. Effect of Silica Fume Addition and Initial Thermal Treatment on Compressive Strength

The strength development of fly ash (Class F)-based geopolymer paste is very slow [22]; thus, early strength is also low. To improve the low strength, previous research has increased the fineness of the fly ash, included additional additives such as SF or OPC, or applied a high temperature curing [19,24,38,39,40]. The reported 28 days compressive strength of the pulverized alkali-activated fly ash based paste is 20–23 MPa [38]. The addition of 5 wt % of OPC into a geopolymer system is also effective for increasing the strength up to 50 MPa from 26 MPa [22]. 

In the current study, however, without relying on a highly alkaline activator or the addition of OPC, a suitable strength was obtained by the addition of hydrated lime and SF. A pozzolanic material like fly ash reacts with water and Ca(OH)_2_ under ambient conditions [8]. Due to this pozzolanic reaction, F+L amb reached 8 MPa of compressive strength at 28 days. The heat of hydration (Figure 5) also confirmed this chemical reaction. Previous research on fly ash with natural hydraulic lime has shown 7 MPa of 28 days compressive strength under water curing conditions at 20 °C [7]. However, the compressive strength of these two fly ash systems for use as structural materials are still not as good as normal OPC-based concrete (20–40 MPa at 28 days).

The major contributions of SF for enhancing compressive strength were due to the additional pozzolanic reaction and space-filling effect. At ambient conditions, the major reason for the enhancement was owing to the filling effect since the additional cumulative heat from the SF addition was negligible (Figure 5b). In other words, unreacted fine particles of SF tended to make the microstructure denser, resulting in the reduction of the total porosity or diameter of the pores [41,42]. The total volume of pores, pore size distribution, and proportion of micropores are known to be important for determining the mechanical properties of concrete [43].

As reported previously, a thermal treatment is effective in terms of enhancing the strength of fly ash-based geopolymer concrete [5,43,44]. In this study, the treatment accelerated the pozzolanic reaction of fly ash-based mortars (Figure 5a). As a result, an additional hydration product of calcium silicate hydrate (C-S-H) made the microstructure of the paste denser [42]. This induced an incremental modification of the compressive strength at an early stage (3 days). In the case of F+L+SF stm, most of the Ca(OH)_2_ was consumed (Figure 6), which led to the attainment of its ultimate strength at a very early age. This indicates that even 3 days of steam curing can accelerate the pozzolanic reaction significantly in the given mixture. Furthermore, results for the heat of hydration and strength evolution confirm the early termination of the reaction.

Overall, the significant synergistic effect of space filling and acceleration of the pozzolanic reaction allows for the attainment of a high compressive strength (>50 MPa) at an early age. These two effects are interdependent because additional SF not only fills the pore space that leads to a physical strength gain, but a high surface area of SF allows for an effective supply of reactive SiO_2_ that is a prerequisite for the pozzolanic reaction. This reaction is efficiently accelerated under high temperatures by consuming more Ca(OH)_2_ in the current study. 

### 4.2. Effect of Intensified Pozzolanic Reaction and Space Filling Effect on Pore Structure

The pore structure of the fly ash based mortar became finer by the SF addition and/or the thermal treatment, as confirmed in Figure 7 and Figure 8. It has been confirmed that applying these two modifications are effective at making the microstructure of other system denser, in the case of kaolinite-based mortar [28]. In another study, recycled aggregate concrete with 30% fly ash and 70% OPC was steam cured (65 °C for 4 h) [43]. The steam curing was effective with regard to reducing the average size of the pores. This was explained by the enhancement of the pozzolanic reaction, which improved the microstructure by the additional formation of C-S-H. Similarly, the intensified pozzolanic reaction by the two treatments, which was confirmed by the hydration heat and XRD analyses, certainly led to a denser microstructure.

A finer pore system, as a result of the accelerated pozzolanic reaction by the thermal treatment, does not always lead to a lower total porosity (Figure 7). However, the filling effect by the unreacted SF decreased the total porosity of the mortar by reducing the macro pore (compare F+L amb and F+L+SF amb). Thus, based on experimental results, it can be found out that the addition of SF is more effective for reducing the total porosity of the fly ash based mortar than applying the thermal treatment.

### 4.3. Drying Shrinkage Depending on Weight Loss and Total Porosity

Drying shrinkage, which is one of the major causes that induce cracks in concrete structures [45], can be simply defined by a volumetric change due to free water evaporation [36]. Therefore, this volumetric change is proportional to the weight loss from the water evaporation [46]. Additionally, the mechanism of drying shrinkage is closely related to the internal pore structure [36,47]. The Kelvin–Laplace equation (Equation (1)) is used to build a relationship between the capillary pressure ($\sigma_{cap}$) and diameter of the meniscus (R)

(1) $$\sigma_{cap}=\frac{2\gamma }{R}.$$

The symbol, γ, in Equation (1) is the constant for the surface tension of water (0.073 N/m). The rates of weight loss and drying shrinkage occurred rapidly in both ambient-cured samples (F+L amb and F+L+SF amb). With the addition of SF (F+L+SF amb), the total weight loss was decreased. This can be explained by more consumption of water by a promoted pozzolanic reaction and adsorption on the surface of the SF particles. In this study, the latter should dominantly contribute to the mitigation of weight loss, since there was no remarkable intensification in hydration reaction by the addition of SF under the ambient condition (Figure 5). Nevertheless, the final drying shrinkage of F+L+SF amb was twice that of F+L amb (Figure 9). This may indicate that water evaporation (weight loss) is not the only cause of drying shrinkage in the current system. In the case of hydrated cement paste, the critical size of the capillary pores for drying shrinkage is known to be less than 50 nm [48]. Based on the mercury intrusion porosimetry results in Figure 7, the following can explain this phenomenon: whereas the total porosity and portion of large pores (100–1000 nm) are less, the percentage of capillary pores is larger in F+L+SF amb. This can lead to a larger drying shrinkage rate even with a smaller weight loss.

In the thermally treated samples (F+L stm and F+L+SF stm), both weight loss and drying shrinkage were less severe and not converged (Figure 9). The obvious reason for the mitigation of the weight loss at 91 days is due to an increase in the consumption of water due to the accelerated pozzolanic reaction during the thermal treatment (Figure 5). This indicates that the total amount of available free water for future drying shrinkage is less at the time of measurement of drying shrinkage. On the other hand, the weight loss of F+L stm at 91 days is 3 times that of F+L+SF stm. However, they show a similar drying shrinkage value (Figure 9). This indicates that similar water evaporation can cause a 3 times greater degree of drying shrinkage. This can be also explained by Equation (1). In a narrow circular tube (<40 nm of radius), the degree of capillary tension ($\sigma_{cap}$) in the pore fluid is inversely proportional to the Kelvin radius (R) [49]. Therefore, in the range of pore diameters less than 80 nm, the pore size in F+L+SF stm is certainly smaller than that in F+L stm, which leads to more induced stress for drying shrinkage.

## 5. Conclusions

Without relying on a high-pH alkaline activator or using OPC, a new high strength fly ash-based mortar was successfully developed. This was possible due to an intensified pozzolanic reaction under initial thermal treatment and space-filling effect. Various mechanical, volumetric, hydration, and microstructural properties of the mortar were investigated as a function of the addition of SF and the treatment.

The addition of SF into the fly ash and hydrated lime based system caused an additional pozzolanic reaction and space-filling effect. In addition, the thermal treatment accelerated the pozzolanic reaction and made the microstructure even denser. When they were applied together, there was a significant synergistic effect, i.e., close to the ultimate strength was obtained at a very early age due to the acceleration and intensification of the pozzolanic reaction by SF. This reduced the total porosity and made the pore system finer, resulting in the improvement of compressive strength, flexural strength, and the modulus of elasticity.

When either SF was added or the thermal treatment was applied in a sample, hydration heat was increased and the intensity of the Ca(OH)_2_ peak decreased. However, without the initial thermal treatment, the SF particles mainly played a role as unreacted fillers; thus, their contribution to the reaction or material performance was negligible.

Samples cured at ambient conditions experienced rapid drying shrinkage and weight loss. While these showed convergence between the ages of 7 and 14 days, the samples with the thermal treatment showed slower and lower development of both drying shrinkage and weight loss and did not converge up to 91 days. This is due to the early consumption of available free water by the accelerated pozzolanic reaction. In addition, between the two thermally treated samples, similar drying shrinkage occurred up to 91 days, whereas the weight loss of the sample with SF was only 1/3 that without SF. This is due to the higher volumetric portion of capillary pores in the sample with SF, which leads to a higher capillary stress for drying shrinkage. 

## Figures and Tables

**Table 1:** Oxide compositions of used raw materials (wt %).

Chemical Composition	SiO_2_	Al_2_O_3_	Fe_2_O_3_	CaO	K_2_O	Na_2_O	MgO	P_2_O_5_	TiO_3_	MnO	LOI ^1^	Total
**Fly Ash**	61.7	18.7	5.56	3.99	1.25	1.57	1.18	0.36	0.86	0.066	4.04	99.97
**Hydrated Lime**	0.57	0.54	0.14	74.51	0.12	0.01	1.12	0.01	0.01	0.004	23.00	100.03
**Silica Fume**	96.90	0.29	0.15	1.54	0.64	0.16	0.18	0.05	0.01	0.03	0.02	99.97

^1^ Loss on ignition.

**Table 2:** Mix proportions of fly ash-based mortar.

Samples Name	Curing Program	Water/Binder Ratio	Total Binder (%)	Silica Fume/Binder Ratio	Super Plasticizer/Binder Ratio	Fine Aggregate/Binder Ratio
			Fly Ash:Hydrated Lime			
F+L amb	Ambient curing	0.25	70:30	-	0.03	3
F+L+SF amb				0.15		
F+L stm	Ambient curing including 72 h of steam curing			-		
F+L+SF stm				0.15		
